# An Imidazoline 2 Receptor Ligand Relaxes Mouse Aorta *via* Off-Target Mechanisms Resistant to Aging

**DOI:** 10.3389/fphar.2022.826837

**Published:** 2022-05-12

**Authors:** Francesc Jiménez-Altayó, Anna Cabrera, Andrea Bagán, Lydia Giménez-Llort, Pilar D’Ocon, Belén Pérez, Mercè Pallàs, Carmen Escolano

**Affiliations:** ^1^ Department of Pharmacology, Therapeutics and Toxicology, School of Medicine, Universitat Autònoma de Barcelona, Barcelona, Spain; ^2^ Institut de Neurociències, Universitat Autònoma de Barcelona, Barcelona, Spain; ^3^ Laboratory of Medicinal Chemistry (Associated Unit to CSIC), Department of Pharmacology, Toxicology and Medicinal Chemistry, Faculty of Pharmacy and Food Sciences, Institute of Biomedicine (IBUB), University of Barcelona, Barcelona, Spain; ^4^ Department of Psychiatry and Forensic Medicine, School of Medicine, Universitat Autònoma de Barcelona, Barcelona, Spain; ^5^ Department of Pharmacology, School of Medicine, Universidad de Valencia, Burjassot, Spain; ^6^ Estructura de Recerca Interdisciplinar en Biotecnologia i Biomedicina (ERI BIOTECMED), Universidad de Valencia, Valencia, Spain; ^7^ Pharmacology Section, Toxicology and Medicinal Chemistry, Faculty of Pharmacy and Food Sciences, Institut de Neurociències, University of Barcelona, Barcelona, Spain

**Keywords:** imidazoline receptor 2 ligands, mouse aorta, endothelium-independent vasodilatation, vascular aging, endothelial dysfunction, potassium and calcium ion channels, L-type voltage-gated Ca^2+^ channels, K_ATP_ channels

## Abstract

Imidazoline receptors (IR) are classified into three receptor subtypes (I_1_R, I_2_R, and I_3_R) and previous studies showed that regulation of I_2_R signaling has neuroprotective potential. In order to know if I_2_R has a role in modulating vascular tone in health and disease, we evaluated the putative vasoactive effects of two recently synthesized I_2_R ligands, diethyl (1RS,3aSR,6aSR)-5-(3-chloro-4-fluorophenyl)-4,6-dioxo-1-phenyl-1,3a,4,5,6,6a-hexahydropyrrolo[3,4-c]pyrrole -1-phosphonate (B06) and diethyl [(1-(3-chloro-4-fluorobenzyl)-5,5-dimethyl-4-phenyl-4,5-dihydro-1H-imidazol-4-yl]phosphonate] (MCR5). Thoracic aortas from Oncins France 1 (3- to 4-months-old) and C57BL/6 (3- to 4- and 16- to 17-months-old mice) were mounted in tissue baths to measure isometric tension. In young mice of both strains, MCR5 induced greater relaxations than either B06 or the high-affinity I_2_R selective ligand 2-(2-benzofuranyl)-2-imidazoline (2-BFI), which evoked marginal responses. MCR5 relaxations were independent of I_2_R, as IR ligands did not significantly affect them, involved activation of smooth muscle K_ATP_ channels and inhibition of L-type voltage-gated Ca^2+^ channels, and were only slightly modulated by endothelium-derived nitric oxide (negatively) and prostacyclin (positively). Notably, despite the presence of endothelial dysfunction in old mice, MCR5 relaxations were preserved. In conclusion, the present study provides evidence against a functional contribution of I_2_R in the modulation of vascular tone in the mouse aorta. Moreover, the I_2_R ligand MCR5 is an endothelium-independent vasodilator that acts largely *via* I_2_R-independent pathways and is resistant to aging. We propose MCR5 as a candidate drug for the management of vascular disease in the elderly.

## Introduction

Aging is a major risk factor in developing vascular disease and, consequently, strategies for achieving healthy vascular aging are encouraged. Endothelial dysfunction is defined as an impairment of endothelium-dependent vasodilation, a phenomenon that is frequently found in the aging vasculature (Ungvari et al., 2018). The endothelium is highly sensitive to changes in physical and chemical environmental conditions. Physiological levels of some endothelium-derived vasoactive mediators, such as NO and prostanoids, are crucial for normal endothelial function, and thus, their imbalance in situations of, for example, oxidative and nitrosative stress, could result in vascular dysfunction (El Assar et al., 2012). To address this problem, strategies usually seek to recover physiological levels of endothelium-derived mediators. Nevertheless, alternative approaches such as to potentiating endothelium-independent vasodilation could provide a complementary route to circumvent endothelial dysfunction.

Imidazoline receptors (IR) were identified around 40 years ago, but their molecular identity is not known. They recognize ligands containing an imidazoline nucleus or structurally related moiety (Bousquet et al., 1984; Ernsberger et al., 1987; Ernsberger et al., 1990). Subsequent studies classified these binding sites into three receptor subtypes (I_1_R, I_2_R, and I_3_R) according to different functional and binding studies (Michel and Ernsberger, 1992; Chan et al., 1994). The I_2_R selective ligand CR4056 has entered clinical trials to treat chronic pain (Ferrari et al., 2011; Li, 2017). In addition, stimulation of I_2_R has shown neuroprotective potential in stroke (Zhang et al., 2020). but few studies have focused on the potential effects of vascular I_2_R modulation. Some of them have found a link between I_2_R, vascular relaxation, and antihypertensive effects (Mar et al., 2013; Chen et al., 2014).

Agmatine, the putative endogenous non-selective IR ligand (Piletz et al., 1995; Santhanam et al., 2007; Mar et al., 2013; Chen et al., 2014) induces relaxation mediated by activation of I_2_R and opening of ATP-sensitive K^+^ (K_ATP_) channels in the aorta of Wistar (Chen et al., 2014) and spontaneously hypertensive (Mar et al., 2013) rats. In contrast, I_2_R are not involved in agmatine-induced responses in the Sprague-Dawley rat aorta, which are mediated by NO, small conductance Ca^2+^-activated K^+^ channels, K_ATP_ and inward-rectifying K^+^ channels (Santhanam et al., 2007). The non-selectivity of agmatine may explain, at least partly, discrepancies in its mechanism of action across rat strains, as well as its multi-target effects (Santhanam et al., 2007; Mar et al., 2013; Chen et al., 2014). However, this evidence may also suggest that IR ligands may activate multiple pathways in the vasculature different from I_2_R, and these actions could contribute to their therapeutic effects. On the whole, modulation of I_2_R activity may have potentially interesting clinical applications in vascular disease. Notably, IR research has embarked on its second youth after recent evidence on the therapeutic potential of newly synthesized selective I_2_R ligands as neuroptotective agents (Ferrari et al., 2011; Abás et al., 2017; Griñán-Ferré et al., 2019; Abás et al., 2020; Vasilopoulou et al., 2020; Rodriguez-Arévalo et al., 2021; Vasilopoulou et al., 2021). These new tools may aid in identifying the relevance of I_2_R in vascular health and disease.

In the present study, we aimed to evaluate the potential vasoactive effects of two recently synthesized I_2_R ligands that belong to different chemical classes, diethyl [(1-(3-chloro-4-fluorobenzyl)-5,5-dimethyl-4-phenyl-4,5-dihydro-1H-imidazol-4-yl] phosphonate] (MCR5; Abás et al., 2017) and diethyl (1RS,3aSR,6aSR)-5-(3-chloro-4-fluorophenyl)-4,6-dioxo-1-phenyl-1,3a,4,5,6,6a-hexahydropyrrolo[3,4-c]pyrrole-1-phosphonate) (B06; Abás et al., 2020). The results show that MCR5 is the I_2_R compound that induces greater relaxations in the mouse aorta of two different mice strains. Notably, the high affinity I_2_R selective ligand 2-(2-benzofuranyl)-2-imidazoline (2-BFI) (Hudson et al., 1997) evoked marginal relaxations and agmatine did not relax. Thus, we confirmed that MCR5 relaxations are largely independent of I_2_R activation, are only slightly modulated by endothelium-derived factors, and are mostly mediated through activation of smooth muscle K_ATP_ and inhibition of L-type voltage-gated Ca^2+^ channels. Remarkably, we show that MCR5 relaxations are preserved in both endothelium-denuded arteries and in old mice despite the presence of endothelial dysfunction. We suggest that MCR5 could be used as a novel agent for treating vascular disease in the elderly.

## Materials and Methods

### Animals

Fifty-two male Oncins France 1 (OF1) non-consanguineous mice of 3- to 4-months of age, and thirty-seven (24 male/ 13 female) C57BL/6 consanguineous mice of 3- to 4- (*n* = 20) and 16- to 17- (*n* = 17) months of age were obtained from Charles River (Sant Cugat del Vallès, Spain). All animals were housed at the animals’ facility under constant temperature (20 ± 2°C) and humidity conditions, 12:12 h dark/light cycle, and provided with *ad libitum* food and water. All procedures were performed in accordance with Spanish legislation on “Protection of Animals Used for Experimental and Other Scientific Purposes” and the EU Council directive (2010/63/EU). The experiments were approved by the Ethics Committee of the Universitat Autònoma de Barcelona (approval code: FJA-eut/01).

### Preparation of Isolated Aortic Rings

Mice were euthanized by decapitation under anesthesia (4% isoflurane mixed with 0.8 L/min O_2_). Descending thoracic aorta was removed and placed in oxygenated (95% O_2_, 5% CO_2_) ice-cold Krebs-Henseleit solution (KHS; NaCl 112 mM, KCl 4.7 mM, CaCl_2_ 2.5 mM, KH_2_PO_4_ 1.2 mM, MgSO_4_ 1.2 mM, NaHCO_3_ 25 mM and glucose 11.1 mM). Afterward, fat surrounding the arteries was quickly removed, and aortas were cut into 2 mm segments to analyze vascular reactivity.

### Evaluation of Aortic Reactivity

Aortic segments were mounted onto a four-channel wire myograph (model 620M; Danish Myo Technology, Aarhus, Denmark) under isometric conditions, filled with ice-cold oxygenated KHS (5 ml), according to the protocol previously described (Muntsant et al., 2021). Briefly, aortic rings were left to equilibrate for 30 min at 37°C and were then stretched gradually to a basal tension of 14.7 mN (OF1 mice) or 6 mN (C57BL/6 mice; Chung et al., 2007). Optimal tension in OF1 mice was assessed in preliminary experiments, which showed that 14.7 mN produced good contraction and relaxation responses. After a 45-min stabilization period, arterial segments were exposed twice to a potassium-enriched KHS (containing 100 mM of KCl) to assess tissue viability. After several washes, aortic rings were left to equilibrate for 30 min and were then pre-contracted with 9,11-Dideoxy-11α,9α-epoxymethanoprostaglandin F_2α_ (U46619), a thromboxane A_2_ stable analog, phenylephrine (Phe), an alpha 1 adrenergic receptor agonist, or 5-hydroxytryptamine (5-HT), a 5-HT receptor agonist, to reach around 70–100% of the contraction induced by KCl (100 mM). When precontraction reached a plateau, endothelium-dependent relaxations were assessed by adding cumulative concentrations (0.001–100 µM) of acetylcholine (ACh). After several washes and a resting period of 30 min, concentration-response curves (0.1–30 µM) to the non-selective IR ligand agmatine, and the I_2_R selective ligands 2-BFI (Hudson et al., 1997), B06 (Abás et al., 2020), and MCR5 (Abás et al., 2017) were performed. The maximum concentrations of these compounds that we could add in the bath were limited by the maximum vehicle (dimethyl sulfoxide; DMSO) concentration (0.2%) that did not produce any effect *per se* (results not shown). In a parallel set of experiments, concentration-response curves (0.001–1 mM) to the NO donor sodium nitroprusside or to ACh (0.001–100 µM) in the absence (control) or presence of MCR5 (1 and 3 µM)were performed.

In experiments aimed to assess the contribution of the endothelium on MCR5-induced relaxations, the endothelium was gently removed using a plastic cannula. Aortic segments were considered endothelium-denuded if relaxation to ACh was below 25%.

Before adding the inhibitors for mechanistic studies, preliminary experiments showed the reproducibility of two consecutive MCR5 relaxation curves (Supplementary Figure S1). Therefore, the study of MCR5 mechanisms of action was performed using the first curve as an internal control. The second curve was performed after preincubation (30 min) with the I_2_R ligand 2-(4,5-dihydro-1H-imidazol-2-yl) quinoline (BU224; 10 μM; Nutt et al., 1995) and the I_2_R and I_1_R antagonist idazoxan (10 μM; Coupry et al., 1987), the selective α_2_-adrenoceptor antagonist yohimbine (1 µM), the non-selective nitric oxide synthase inhibitor Nω-nitro-l-arginine methyl ester (L-NAME; 300 µM), the non-selective COX inhibitor indomethacin (10 µM), the inwardly rectifying potassium channel blocker BaCl_2_ (1 mM), the large and small conductance Ca^2+^ -activated K^+^ channels blockers, respectively, charybdotoxin (0.1 µM) and apamin (0.1 µM), the specific blocker of voltage-gated K^+^ channels 4-aminopyridine (1 mM), the ATP-sensitive K^+^ channels blocker glibenclamide (30 µM), or the Na^+^/K^+^-ATPase inhibitor ouabain (1 µM).

In experiments to study the contribution of voltage-gated Ca^2+^ channels, aortic rings were: 1) pre-contracted with KCl (100 mM) before performing concentration-response curves to MCR5; 2) contracted with KCl (8–300 mM) in the absence (control) or presence of the L-type Ca^2+^ channel blocker nifedipine (10 µM) or MCR5 (10 and 30 µM); 3) contracted with U46619 (0.001–1 µM) in the absence (control) or presence of nifedipine (10 µM) or MCR5 (10 and 30 µM); and 4) contracted with Bay K8644 (1 µM) in the absence (control) or presence of MCR5 (10 and 30 µM).

### Measurement of Prostanoid Production

The release of prostanoids from aortic segments was determined by enzyme-linked immunosorbent assays (ELISA; Cayman Chemical, Ann Arbor, MI), as reported (Costa et al., 2019). Levels of prostacyclin (PGI_2_) and thromboxane A_2_ (TXA_2_) stable metabolites 6-Keto-PGF_1α_ and TXB_2_, respectively, were assessed in the KHS collected in resting conditions (control) or after performing concentration-response curves to MCR5 in the absence or presence of indomethacin (10 µM). Samples were kept at room temperature for 30 min for complete hydrolysis of PGI_2_ and TXA_2_ into their stable metabolites. Prostanoid levels were assessed in duplicate and calculated according to the ELISA kit manufacturer instructions.

### Statistical Analysis

Statistical analysis was performed with GraphPad Prism version 8.3 (San Diego, California, United States) software. Results are expressed as mean ± SEM (standard error of the mean) of the number (*n*) of animals in each group (shown in figure legends). Vasodilator responses to ACh, 2-BFI, B06, and MCR5 are expressed as a percentage of the tone generated by U46619, Phe, 5-HT, or KCl (100 mM) pre-contractions. Sigmoid curve fitting (variable slope) was performed by non-linear regression to obtain maximal responses (*E*_*max*_) and sensitivity (*pEC*_*50*_) in the vasoconstrictor responses curves to KCl and U46619. Statistical analysis was carried out using one-way ANOVA or Kruskal-Wallis with Tukey’s or Dunn’s *post hoc* test, respectively, for a single factor. In case of two factors, two-way ANOVA corrected for multiple comparisons using Bonferroni post-test was used. Statistical significance was set as *p* ≤ 0.05.

### Chemicals

Selective I_2_R ligands B06 and MCR5 were provided by Dr. Carmen Escolano from the laboratory of Medicinal Chemistry (Faculty of Pharmacy and Food Sciences, Universitat de Barcelona, Barcelona, Spain), and 2-BFI was purchased from Tocris (Bio-Techne R&D Systems S. LU, Madrid, Spain). ACh, sodium nitroprusside, U46619, Phe, 5-HT, BU224, idazoxan, L-NAME, indomethacin, 4-aminopyridine, glibenclamide, BaCl_2,_ ouabain, Bay K8644, and agmatine were purchased from Merck (KGaA, Darmstadt, Germany). ACh, sodium nitroprusside, Phe, 5-HT, 4-aminopyridine, BaCl_2_, ouabain, Bay K8644, and agmatine were diluted in distilled water; 2-BFI, B06, MCR5, U46619, BU224, idazoxan and glibenclamide were diluted in DMSO (final bath concentration ≤ 0.2%); L-NAME was diluted in KHS; and indomethacin was diluted in 2% NaHCO_3_.

## Results

### MCR5 Induces Greater Relaxations Than B06 and 2-BFI

**FIGURE 1:**
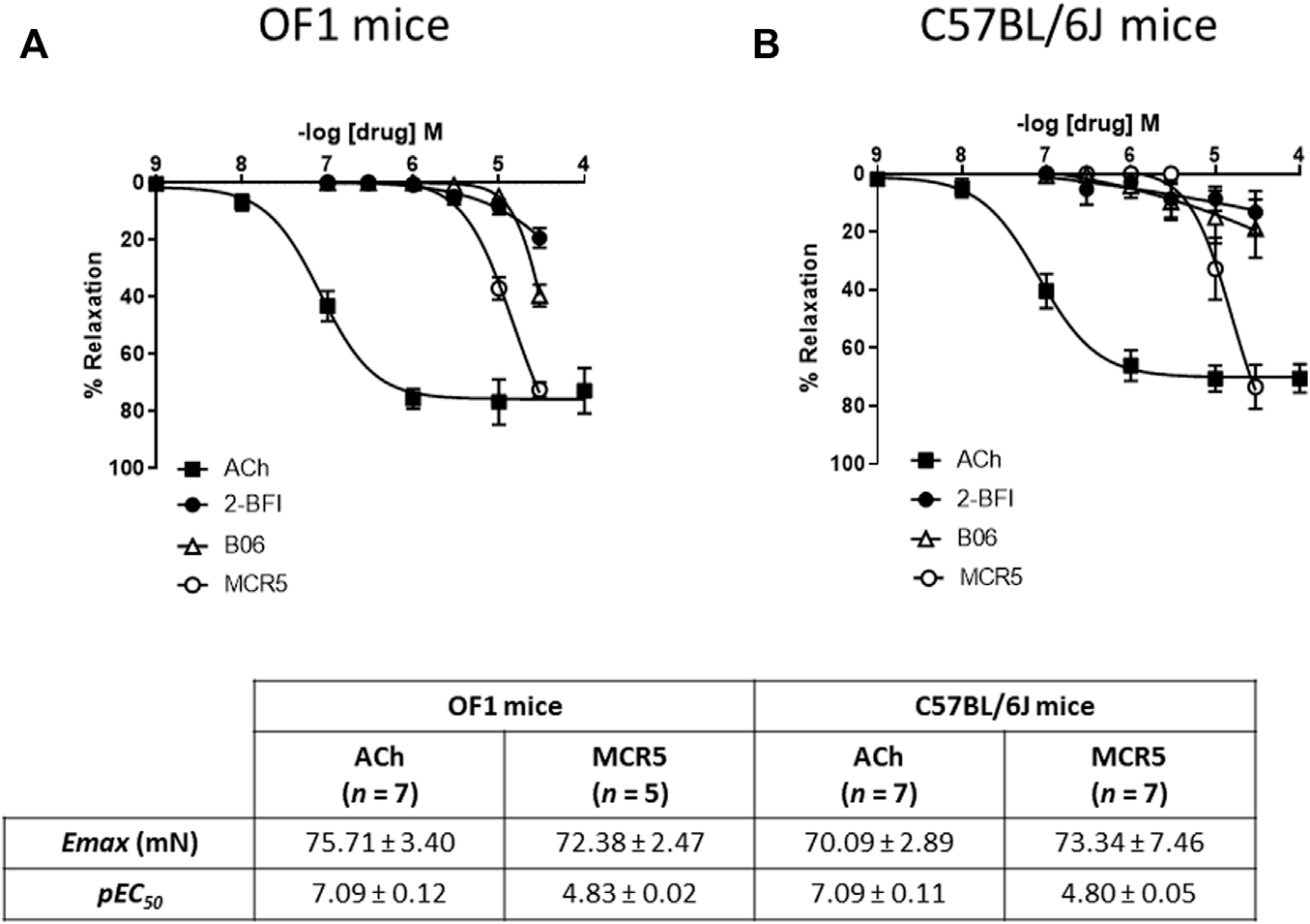
Comparison of I_2_R ligands relaxations. Concentration-response curves to acetylcholine (ACh) and I_2_R ligands in U46619-pre-contracted aortic rings from young OF1 **(A)** and C57BL/6J **(B)** mice. Maximum response (*E*_*max*_) and potency (*pEC*_*50*_) of ACh and MCR5 relaxations are shown at the bottom. Results are mean ± SEM from *n* = 7 (ACh), 5-7 (2-BFI), 6 (B06), 5-7 (MCR5) mice.

Neither of the compounds was able to induce aortic contractions. Figure 1 shows concentration-dependent relaxations to ACh (an endothelium-dependent agonist used as a relaxation control) *vs.* three structurally different I_2_R ligands in U46619-pre-contracted thoracic aortas from young OF1 and C57BL/6 mice. The results show that MCR5 induces greater relaxations, as compared to B06 and 2-BFI, following a similar pattern in both strains. Note that relaxations to 2-BFI, an I_2_R prototype ligand, were the lowest ones. Although MCR5 relaxations were less potent than ACh relaxations, maximum responses were similar (Figure 1).

### MCR5 Relaxations Are Largely Non-mediated by I_2_R

**FIGURE 2:**
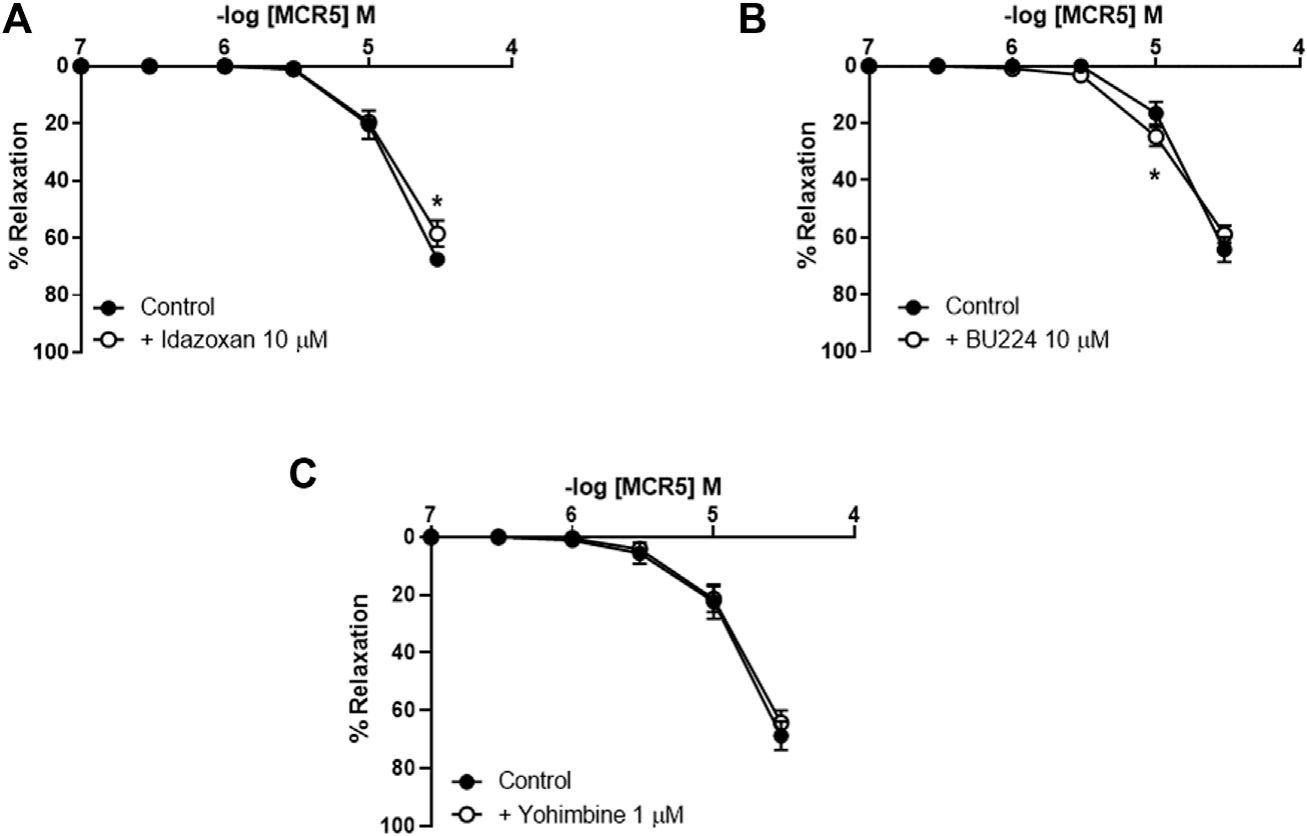
Effects of I_2_R modulators and antagonism of α_2_-adrenoceptors on MCR5 relaxations. Concentration-response curves to MCR5 in U46619-pre-contracted aortic rings from young OF1 mice in the absence (control) or presence of the I_1_R and I_2_R ligand idazoxan (10 µM) **(A)** the I_2_R ligand BU224 (10 µM) **(B)**, or the α_2_-adrenoceptor antagonist yohimbine (1 µM) **(C)**. Results are mean ± SEM from *n* = 7 **(A)**, 8 **(B)**, and 6 **(C)**. ∗*p* < 0.05 by two-way repeated measures ANOVA with Bonferroni’s *post hoc* test.

Incubation with two different I_2_R ligands, idazoxan (I_1_R and I_2_R ligand; 10 μM; Figure 2A) and BU224 (I_2_R ligand; 10 μM; Figure 2B), only slightly decreased or increased, respectively, MCR5 relaxations. These results suggest that MCR5 relaxations are largely independent of I_2_R signaling. Of note, agmatine did not evoke any response in U46619-precontracted aortas from OF1 mice (results not shown). Newly synthesized imidazoline compounds are typically screened according to their ability to activate α_2_-adrenoceptors (Bousquet et al., 2020). We subsequently studied the effect of the selective α_2_-adrenoceptor antagonist yohimbine, which did not affect MCR5 relaxations (Figure 2C).

### MCR5 Relaxations Are Higher in KCl Pre-Contracted Vessels

**FIGURE 3:**
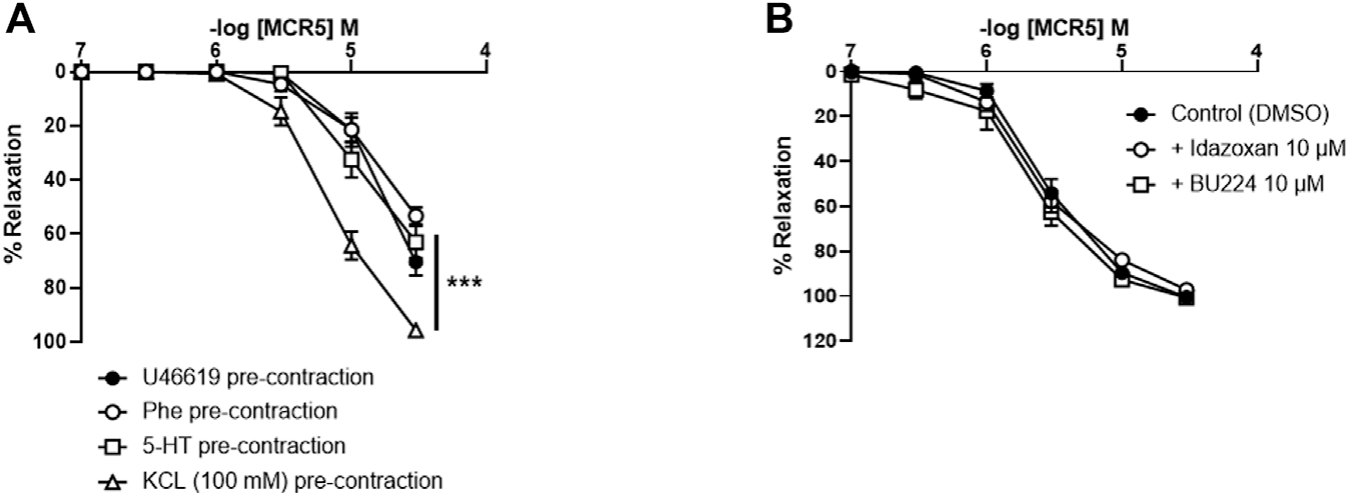
Effects of different pre-contractile agonists on MCR5 relaxations and influence of I_2_R modulation. Concentration-response curves to MCR5 in U46619-, phenylephrine (Phe)-, 5-hydroxytryptamine (5-HT)-, and KCL (100 mM)-pre-contracted aortic rings from young OF1 mice **(A)**. Concentration-response relaxation curves to MCR5 in KCl (100 mM)-pre-contracted aortic rings in the absence (Control) or presence of idazoxan (10 µM) and BU224 (10 µM) **(B)**. Results are mean ± SEM from *n* = 5 **(A)** and 3-4 **(B)** mice. ∗∗∗*p* < 0.001 by two-way repeated measures ANOVA with Bonferroni’s *post hoc* test.

Concentration-response curves to MCR5 were similar regardless of the use of Phe, 5-HT, or U46619 as precontractile agonists (Figure 3A). Only relaxations to 30 µM MCR5 were significantly lower (*p* < 0.001) in Phe- (53.26 ± 3.09%, *n* = 5) *vs.* U46619- (70.47 ± 4.83%, *n* = 5) pre-contracted arteries. Notably, MCR5 responses were markedly greater in KCl (100 mM)-pre-contracted arteries (Figure 3A). Neither idazoxan (10 µM) nor BU224 (10 µM) altered these relaxations (Figure 3B), confirming that I_2_R are not significantly involved in MCR5 responses.

### MCR5 Blocks Smooth Muscle L-Type Voltage-Gated Ca^2+^ Channels

**FIGURE 4:**
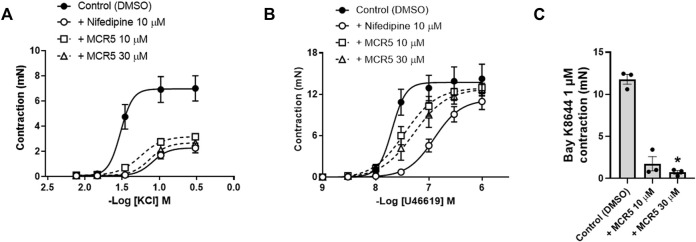
Influence of MCR5 on smooth muscle voltage-gated Ca^2+^ channels. Concentration-response contraction curves to KCl **(A)** and U46619 **(B)** in aortic rings from young OF1 mice in the absence (control) or presence of the L-type voltage-gated Ca^2+^ channel blocker nifedipine (10 µM) or MCR5 (10 and 30 µM). **(C)** Contractions to the L-type voltage-gated Ca^2+^ channel activator Bay K8644 (1 µM) in the absence (control) or presence of MCR5 (10 and 30 µM). Results are mean ± SEM from *n* = 4 **(A,B)** or *n* = 3 **(C)** mice. ∗*p* < 0.05 by Kruskal-Wallis with Dunn’s *post hoc* test.

In view of the increased MCR5 relaxations in KCl pre-contracted aortas, we studied the potential role of the compound in blocking smooth muscle voltage-gated Ca^2+^ channels (Figure 4). Incubation with MCR5 10 or 30 µM caused a significant decrease in maximal contractions to KCl similar to that induced by the L-type Ca^2+^ channel blocker nifedipine (10 µM) (Figure 4A; Table 1). In addition, U46619 contractions were not significantly reduced by nifedipine, nor MCR5, but were slightly (*p* = 0.059) shifted to the right after nifedipine and MCR5 (30 µM) incubation (Figure 4B; Table 1). Moreover, MCR5 (30 µM) significantly reduced contractions induced by the L-type Ca^2+^ channel activator Bay K8644 (1 µM) (Figure 4C). Taken together, these results suggest that MCR5 blocks L-type voltage-gated Ca^2+^ channels.

**TABLE 1:** Maximum response (*E*_*max*_) and potency (*pEC*_*50*_) of the concentration–response curves to KCL (*n* = 4) and U46619 (*n* = 4) contractions (mN) in aortic rings from young OF1 mice.

		Control (DMSO)	+ Nifedipine 10 µM	+ MCR5 10 µM	+ MCR5 30 µM
KCl	*E*_*max*_ (mN)	6.96 ± 0.54	2.28 ± 0.22***	3.18 ± 0.19***	2,69 ± 0.30***
	*pEC*_*50*_	1.52 ± 0.12	1.09 ± 0.11 p=0.058	1.25 ± 0.06	1.13 ± 0.12
U46619	*E*_*max*_ (mN)	13.72 ± 0.83	11.22 ± 0.91	12.94 ± 0.91	12.83 ± 1.14
	*pEC*_*50*_	7.69 ± 0.10	6.90 ± 0.08***	7.46 ± 0.10	7.28 ± 0.12 p=0.059

***Versus Control (DMSO) by one-way ANOVA with Tukey’s *post hoc* test.

### MCR5 Relaxations Are Modulated by K_ATP_, Voltage-Gated K^+^ Channels, and the Na^+^/K^+^-ATPase

**FIGURE 5:**
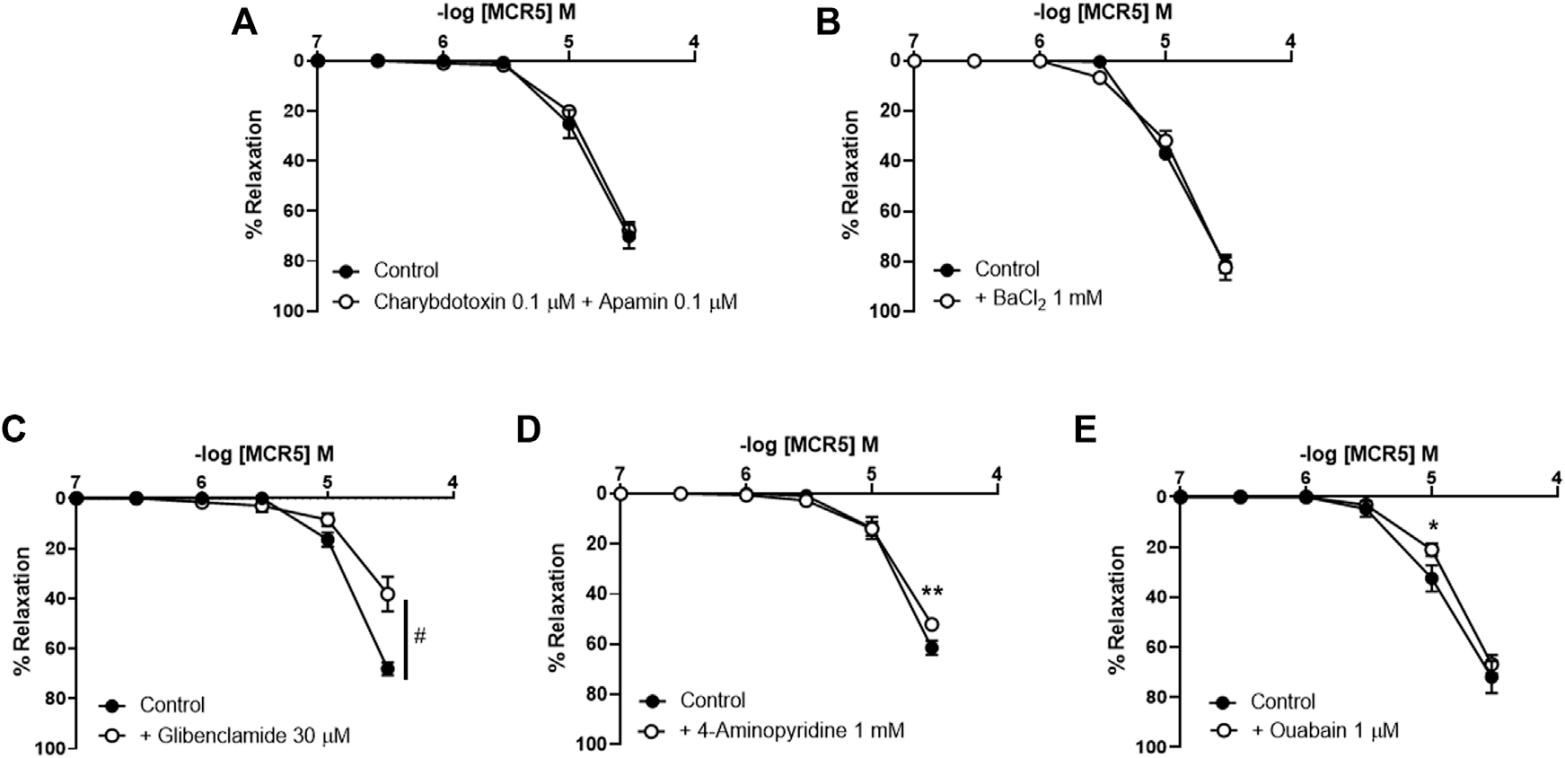
Effects of inhibition of K^+^ channels and the Na^+^/K^+^-ATPase on MCR5 relaxations. Concentration-response curves to MCR5 in U46619-pre-contracted aortic rings from young OF1 mice in the absence (control) or presence of the Ca^2+^-activated K^+^ channels inhibitors charybdotoxin (0.1 µM) and apamin (0.1 µM) **(A)**, the inwardly rectifying K^+^ channel blocker BaCl_2_ (1 mM) **(B)**, the K_ATP_ channel blocker glibenclamide (30 µM) **(C)**, the voltage-gated K^+^ channel blocker 4-aminopyridine (1 mM) **(D)**, and the Na^+^/K^+^-ATPase inhibitor ouabain (1 μM) **(E)**. Results are mean ± SEM from *n* = 6 **(A)**, 5 **(B)**, 7 **(C)**, 6 **(D,E)** mice. ^#^ *p* < 0.05 by two-way repeated measures ANOVA; ∗*p* < 0.05, ∗∗*p* < 0.01 by two-way repeated measures ANOVA with Bonferroni’s *post hoc* test.

To investigate if relaxations were dependent upon K^+^ efflux, we pre-treated vessels with different K^+^ channel inhibitors. Neither inhibition of K_Ca_ channels with charybdotoxin (100 nM) and apamin (100 nM) (Figure 5A), nor inhibition of KIR channels with BaCl_2_ (1 mM) (Figure 5B) modified MCR5 relaxations. In contrast, inhibition of K_ATP_ channels with glibenclamide (30 µM) significantly reduced MCR5 responses (Figure 5C). Voltage-gated K^+^ channels blockade with 4-aminopiridine (1 mM) only slightly decreased maximal relaxations (Figure 5D). The activity of the Na^+^/K^+^-ATPase is another mechanism that contributes to vascular relaxation. Addition of the Na^+^/K^+^-ATPase inhibitor ouabain (1 μM) slightly decreased MCR5 relaxations (Figure 5E).

### MCR5 Relaxations Are Modulated by Endothelial-Derived NO and Prostacyclin

**FIGURE 6:**
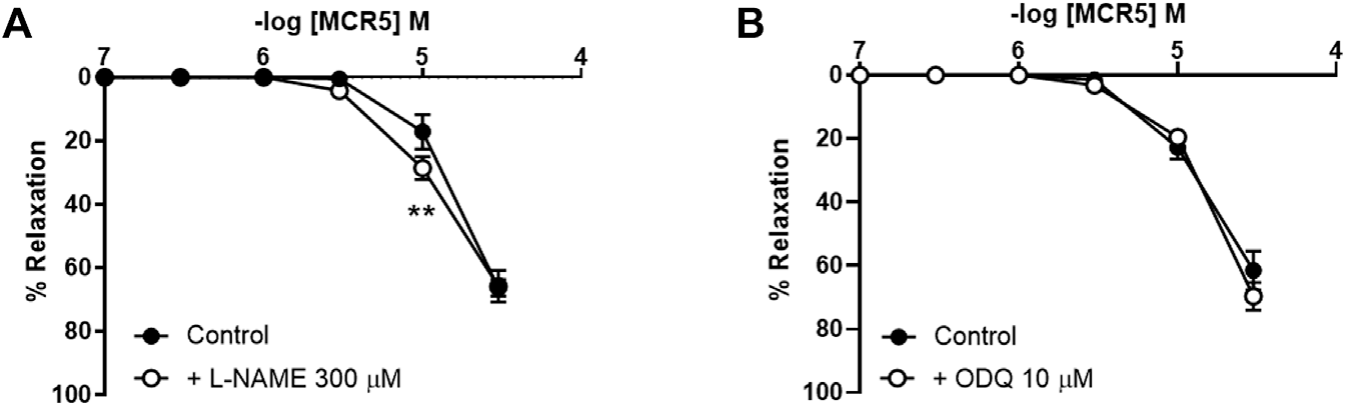
Effects of NO pathway inhibition on MCR5 relaxations. Concentration-response curves to MCR5 in U46619-pre-contracted aortic rings from young OF1 mice in the absence (control) or presence of the NO synthase inhibitor L-NAME (300 µM) **(A)** or the soluble guanylyl cyclase inhibitor ODQ (10 µM) **(B)**. Results are mean ± SEM from *n* = 7 **(A)** and 5 **(B)** mice. ∗∗*p* < 0.01 by two-way repeated measures ANOVA with Bonferroni’s *post hoc* test.

**FIGURE 7:**
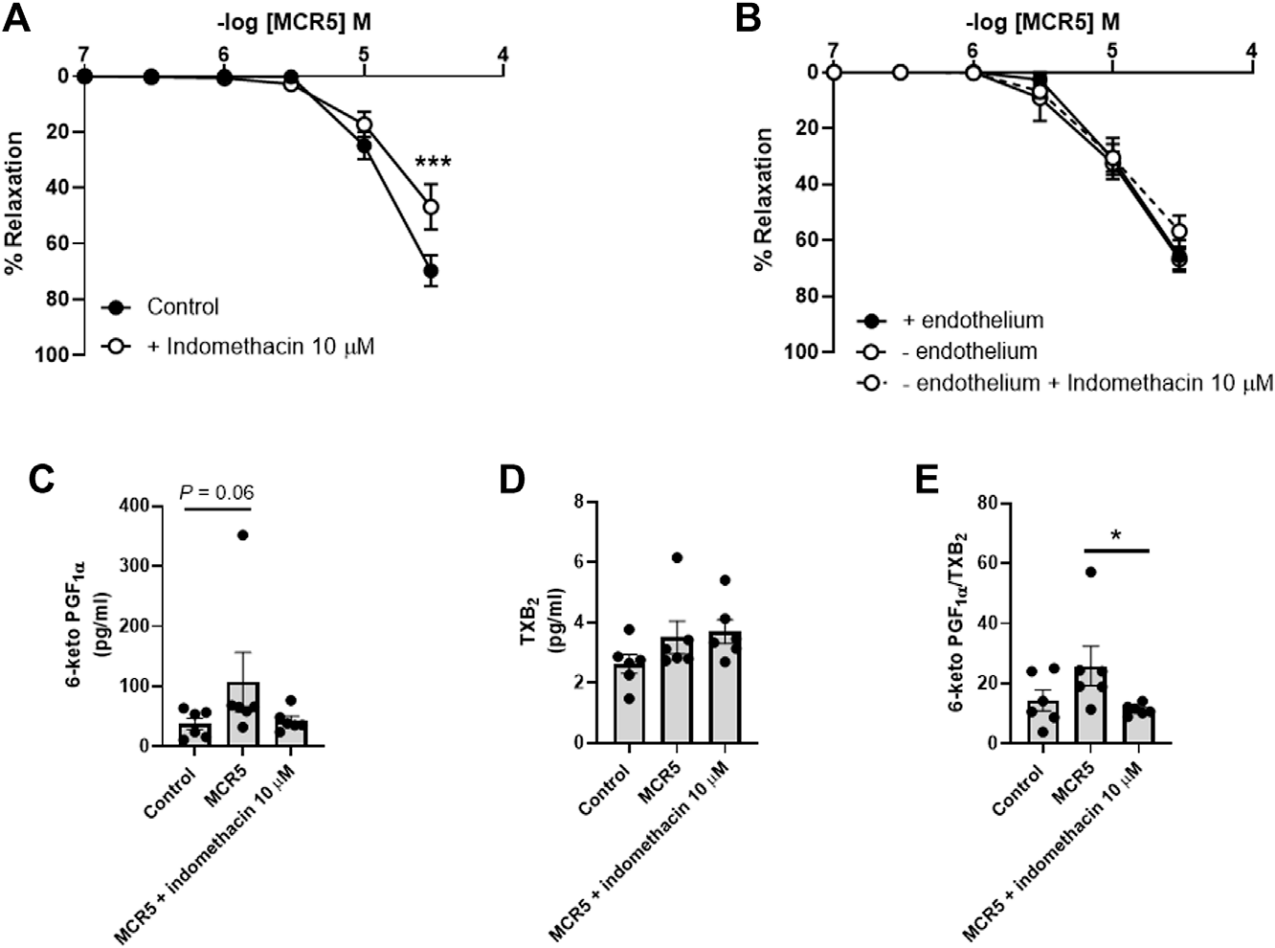
Effects of COX inhibition on MCR5-induced aortic relaxations and prostanoids production. Concentration-response curves to MCR5 in U46619-pre-contracted aortic rings from young OF1 mice in the absence (control) or presence of the COX inhibitor indomethacin (10 µM) **(A)**. Concentration-response curves to MCR5 in endothelium denuded arteries (- endothelium) in the absence or presence of indomethacin **(B)**. Levels of PGI_2_ and TXB_2_ metabolites, 6-ketoPGF_1α_ **(C)** and TXB_2_ **(D)**, respectively, and the 6-ketoPGF_1α_/TXB_2_ ratio **(E)**, in the Krebs-Henseleit solution collected after concentration-response curves to MCR5 in U46619-pre-contracted aortic rings from young OF1 mice. Results are mean ± SEM from *n* = 7 **(A)**, 4–6 **(B)**, and 6 **(C**, **D**, and **(E)**. ∗∗∗*p* < 0.001 by two-way repeated measures ANOVA with Bonferroni’s post hoc test **(A)**; ∗*p* < 0.05 by Kruskal-Wallis with Dunn’s *post hoc* test **(C,E)**.

Inhibition of NO synthesis with L-NAME (300 µM) modestly increased MCR5-induced relaxations (Figure 6A), suggesting a slight negative influence related to NO, which was not mediated by. soluble guanylyl cyclase since ODQ (10 µM), an inhibitor of this enzyme, did not affect MCR5 relaxations (Figure 6B). Non-selective inhibition of COX with indomethacin (10 µM) significantly reduced MCR5 relaxations (Figure 7A). We next explored whether the endothelium could be the source of NOS- and COX-mediated products that affect, negatively or positively, respectively, MCR5 responses. As expected, removal of the endothelium almost abolished ACh vasodilation (Supplementary Figure S2). However, in the same aortic rings, the absence of endothelium did not modify MCR5 relaxations (Figure 7B). The observation that relaxations were unaltered after endothelium removal despite the negative feedback produced by endothelial-derived NO on MCR5 responses, raises the hypothesis that MCR5 releases an endothelium-derived COX-mediated relaxing factor, such as prostacyclin (PGI_2_). Interestingly, endothelium removal abolished the inhibitory effect of indomethacin on MCR5 relaxations (Figure 7B). Prostanoids release from aortic rings was determined in the KHS solution collected after concentration-response curves to MCR5. MCR5 tended (*p* = 0.06) to increase the release of PGI_2_ (Figure 7C), whereas thromboxane (TXA_2_) was not significantly increased (Figure 7D). Remarkably, indomethacin significantly (*p* < 0.05) reduced the PGI_2_/TXA_2_ ratio in the presence of MCR5 (Figure 7E). These results suggest that MCR5 releases endothelial-derived prostacyclin contributing to relaxation.

### MCR5 Relaxations Are Not Impaired in Old Mice Despite the Presence of Endothelial Dysfunction

**FIGURE 8:**
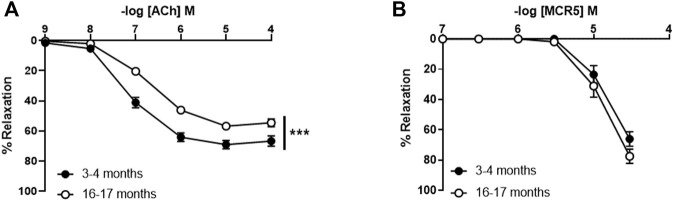
Influence of aging on aortic relaxations. Concentration-response curves to acetylcholine (ACh) in U46619-pre-contracted aortic rings from 3- to 4-month-old (young) and 16- to 17-months-old (old) C57BL/6 mice **(A)**. Concentration-response curves to MCR5 **(B)**. Results are mean ± SEM from *n* = 13–15 **(A,B)** mice. ∗∗∗*p* < 0.001 by two-way repeated measures ANOVA with Bonferroni’s *post hoc* test.

To study the potential relevance of MCR5 in aging-related vasodilator dysfunction, we used aortic rings from young (3–4 months) and old (16–17 months) C57BL/6 mice (Figure 8). We firstly evaluated ACh relaxations, which were significantly impaired in old compared to young mice (Figure 8A). However, endothelium-independent relaxations to the NO donor sodium nitroprusside were not altered in old mice (Supplementary Figure S3), suggesting that direct smooth muscle relaxation is not affected by aging. In addition, these results indicate that impairments of ACh responses in old mice are a result of endothelial dysfunction. Remarkably, relaxations to MCR5 were not affected by aging (Figure 8B).

To know if MCR5, additionally to its endothelium independent vasorelaxant activity, could improve the age-related endothelial dysfunction, we further studied whether concentrations of MCR5 that evoked marginal relaxations (1 or 3 µM) may improve ACh responses. The results show that MCR5 does not modify ACh vasodilations (Supplementary Figure S4).

## Discussion

The development of new strategies to modulate vascular responses is important to discover pharmacological tools to face vascular disease. In the present study, we explored the putative vasoactive properties of selective I_2_R ligands that have previously shown neuroprotective activity in age-related neurodegenerative disorders such as Alzheimer’s disease (Abás et al., 2017; Griñán-Ferré et al., 2019; Abás et al., 2020). The results not only might explain some of the mechanisms involved in neuroprotection, but also might pave the way towards the identification of I_2_R modulatory molecules with therapeutic potential against vascular disease in the elderly. Nevertheless, our findings indicate that I_2_R might not have an important role in the modulation of vascular tone in the mouse aorta.

Previous studies reported that both I_1_R and I_2_R are present in the vasculature (Regunathan et al., 1995; Mar et al., 2013; Sarac et al., 2015). The vascular effects of I_1_R activation have been primarily related to the central regulation of blood pressure (Ernsberger et al., 1995), whereas vascular I_2_R signaling is associated with vasodilation and antihypertensive effects (Mar et al., 2013; Chen et al., 2014). Compounds B06 and MCR5 have been recently recognized as selective I_2_R ligands with neuroprotective potential in Alzheimer’s disease (Abás et al., 2017; Griñán-Ferré et al., 2019; Abás et al., 2020). In the present study, we studied the putative vasoactive actions of these compounds in two different mice strains (OF1 and C57BL/6J). We found that although both compounds cause aortic dilation, MCR5 was the one that induced greater relaxations in both mice strains. Surprisingly, these responses were largely non-mediated by I_2_R signaling. Contrasting results were reported regarding the mechanism of vasodilatation induced by the non-selective IR ligand agmatine across rat strains (Santhanam et al., 2007; Mar et al., 2013; Chen et al., 2014). Here, in the mouse aorta, agmatine did not evoke any response and relaxations induced by the selective I_2_R ligand MCR5 were largely independent of I_1_R, I_2_R and α_2_-adrenoceptors, since idazoxan (I_1_R and I_2_R, but not I_3_R ligand; Coupry et al., 1987) or BU224 (I_2_R ligand; Nutt et al., 1995) only slightly affected them, and yohimbine (α_2_-adrenoceptor antagonist) did not alter them. This argument was further substantiated by the fact that relaxations induced by the I_2_R prototype ligand 2-BFI (Hudson et al., 1997), by B06, another selective I_2_R ligand that belongs to a different chemical class compared to MCR5 (Abas et al., 2020), and by agmatine, a non-selective IR ligand, were minimal or absent providing additional evidence in support of a marginal contribution of I_2_R signalling in MCR5 relaxations in the mouse aorta, at least in the current study experimental conditions. Indeed, these findings raise the question whether I_2_R are expressed in the mouse aorta. Unfortunately, this cannot be answered until IR molecular identity is known.

Potassium channels exert an important contribution to the regulation of vascular tone (Tykocki et al., 2017). The activation of K_ATP_ channels induces hyperpolarization, closing of voltage-gated Ca^2+^ channels and Ca^2+^ influx, and consequently, relaxes smooth muscle. Note that K_ATP_ channels have been consistently involved in agmatine responses in the rat aorta (Santhanam et al., 2007; Mar et al., 2013; Chen et al., 2014) and here, we found that MCR5 relaxation of the mouse aorta was partly mediated *via* activation of K_ATP_, since it was attenuated by glibenclamide. It is proposed that I_3_R receptors represent a binding site present in the Kir6.2 subunit of K_ATP_ channels in pancreatic beta cells (Ishida-Takahashi et al., 1996; Proks and Ashcroft, 1997; Bousquet et al., 2020). We can speculate that MCR5 induces vasodilatation by binding to a putative vascular I_3_R receptor or directly to vascular K_ATP_ channels, activating K^+^ efflux, and promoting membrane hyperpolarization. This change in membrane potential may affect the open probability of voltage-gated Ca^2+^channels, facilitating vasodilatation. The possibility that MCR5 acts through I_3_R remains open, but the lack of previous evidence on the presence and functional role of I_3_R in vessels does not support this hypothesis at this stage.

Participation of other K^+^ channels as Ca^2+^-activated K^+^ channels or inwardly rectifying K^+^ channels can be discarded, as selective inhibitors as charybdotoxin and apamin, or BaCl_2_, respectively, did not alter MCR5 vasodilator activity. Only a minor contribution of voltage-gated K^+^ channels was observed. However, at this stage, we cannot discard that the effect of 4-aminopiridine on MCR5 relaxations might be a consequence of voltage-gated Ca^2+^ channel activation (Wu et al., 2009). In fact, we found that inhibition of voltage-gated Ca^2+^ channels was involved in MCR5 relaxations, an effect that was independent of I_2_R, but might be a consequence of K_ATP_ channels opening related to I_3_R. Voltage-gated Ca^2+^ channels greatly contribute to the control of resting membrane potential in aortic smooth muscle cells (Tammaro et al., 2004; Tykocki et al., 2017). The extent of the inhibitory effect of MCR5 and nifedipine was similar in KCl-induced contraction-response curves, but with some differences in U46619-induced contractions. The main mechanism associated with KCl-induced contractions is the opening of L-type voltage-gated Ca^2+^-channels, which suggests an involvement of this type of channel on MCR5 vasodilatations. Consistently, MCR5 greatly reduced contractions evoked by the L-type voltage-gated Ca^2+^-channel activator Bay K8644. These results agree, at least in part, with previous evidence showing that agmatine blocks L-type voltage-gated Ca^2+^ channels and other types of voltage-gated Ca^2+^ channels in cultured rat hippocampal neurons (Weng et al., 2003; Zheng et al., 2004). However, other mechanisms such as increased reactive oxygen species production, K_ATP_ channel dysfunction and impaired NO-dependent vasodilation contribute to the thromboxane A_2_ analogue U46619 vascular smooth muscle contractions (Santos et al., 2021). This evidence may explain slight differences found between the inhibitory effect of nifedipine and MCR5 on U46619 *versus* KCl concentration-response curves.

Vascular tone is a tightly regulated phenomenon that involves multiple signalling pathways, whose deregulation results in the development of vascular pathology. The ability of MCR5 to induce robust relaxations in endothelium-denuded arteries suggests that this compound mostly relaxes the mouse aorta through endothelium-independent mechanisms. Besides, numerous vasoconstrictors and vasodilators can modulate, either directly or indirectly, smooth muscle ion channel activity and, consequently, vascular tone.

In physiological conditions, endothelial cells regulate vascular tone by producing a negative feedback on vascular smooth muscle contractility (Mohrman and Heller, 2014). An important relaxation mediator produced by endothelial cells is NO, which is synthesized in response to a wide variety of stimuli. In addition, the endothelium regulates basal tone not only through the production of NO, but also through the release of other endothelium-derived relaxing and contracting factors able to influence vascular smooth muscle contraction in a paracrine fashion (Mohrman and Heller, 2014). For instance, prostanoids, lipid mediators produced by COX, are potent endothelium- and smooth muscle-derived vasodilators and vasoconstrictors that can actively modulate vascular tone (Wong et al., 2010). MCR5 vasodilatation was not mediated by NO, since L-NAME did not reduce relaxations, but slightly increased them. The fact that the soluble guanylyl cyclase inhibitor ODQ did not reproduce this effect suggests that the slight NO negative influence on MCR5 relaxations was not mediated by a downstream signaling pathway of this enzyme but could be related to endothelial mechanisms. Non-selective COX inhibition with indomethacin decreased either MCR5 relaxations or MCR5-induced increase in prostacyclin levels. These results demonstrate that MCR5 relaxations are positively modulated by COX activation in the aorta of OF1 mice. Notably, the inhibitory effect of indomethacin was not present after endothelium removal, suggesting that the compound may activate endothelial COX. Altogether, these results provide evidence in support of an opposed modulatory effect of NO and prostacyclin endothelial pools on MCR5 relaxations.

The classical approach to protecting dysfunctional vessels is to administer compounds with anti-oxidant or anti-inflammatory activities, which, among other beneficial effects, are able to restore physiological levels of vasoactive factors. However, some treatments with these compounds can cause adverse effects or their effectiveness have not been demonstrated in the clinical setting (Costa et al., 2021). Interestingly, vasodilation through endothelium-independent mechanisms could also represent a strategy to warrant physiological responses in adverse environmental conditions that have the potential to affect endothelial function, like in those reported in vascular aging (El Assar et al., 2012; Ungvari et al., 2018). Along these lines, endothelium-independent relaxations induced by sodium nitroprusside were preserved in old mice, suggesting that strategies that promote direct smooth muscle relaxation might be more resistant to aging-related vascular dysfunction. Importantly, we found that MCR5 responses were conserved in old mice in spite of impaired ACh relaxations. Therefore, present findings demonstrate that MCR5 relaxations are protected in the setting of endothelial dysfunction, likely because of they are largely independent of endothelium-derived signalling.

The present study has some limitations. First, it is not possible to identify I_2_R in vessels since their molecular identity is unknown; and second, the lack of a direct evidence about the proposed relationship between MCR5 activity and K_ATP_ and L-type voltage-gated Ca^2+^ channels. Finally, it would be interesting to know the putative vasoactive effects of MCR5 in small resistance peripheral and cerebral arteries, which may provide relevant information about the impact of the compound on, for instance, blood pressure or cerebrovascular autoregulation, respectively.

In our hands, MCR5 showed a pleiotropic action related primarily with endothelium independent routes that explained its vascular effects. These actions included activation of smooth muscle K_ATP_ and inhibition of L-type voltage-gated Ca^2+^ channels. Furthermore, vasodilation was slightly modulated by endothelial nitric oxide (negatively) and prostacyclin (positively), effects that compensate each other. Importantly, MCR5 relaxations were preserved either in endothelium-denuded vessels or in old mice, suggesting that this compound may have therapeutic potential in the setting of aging-related endothelial dysfunction. Overall, the present study adds knowledge on the understanding of the effects of I_2_R ligands in vessels and provides evidence against a functional contribution of I_2_R in the modulation of vascular tone in the mouse aorta. MCR5 is a I_2_R ligand with an interesting vasodilator activity largely mediated through I_2_R- and endothelium-independent mechanisms. This vasorelaxant activity is resistant to aging-related dysfunction, suggesting that MCR5 may be an interesting tool for treating vascular disease associated with aging.

## Data Availability

The raw data supporting the conclusion of this article will be made available by the authors, without undue reservation.
