# Supplementary material for: An Imidazoline 2 Receptor Ligand Relaxes Mouse Aorta via Off-Target Mechanisms Resistant to Aging
Source: Front Pharmacol. 2022 May 12;13:826837. doi: 10.3389/fphar.2022.826837 (PMC9133327; doi:10.3389/fphar.2022.826837)
Supplement: Supplementary file 1 [file DataSheet1.PDF]

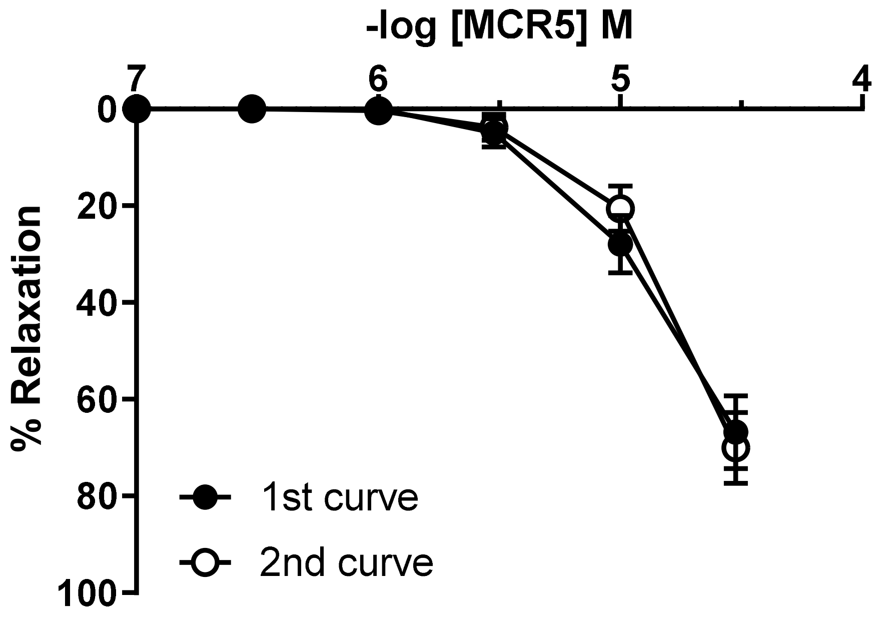

Supplementary Figure 1. Reproducibility of MCR5 relaxations. Consecutive concentration-response curves to MCR5 in U46619-pre-contracted aortic rings from young OF1 mice. Results are mean ± SEM from *n* = 4 mice.

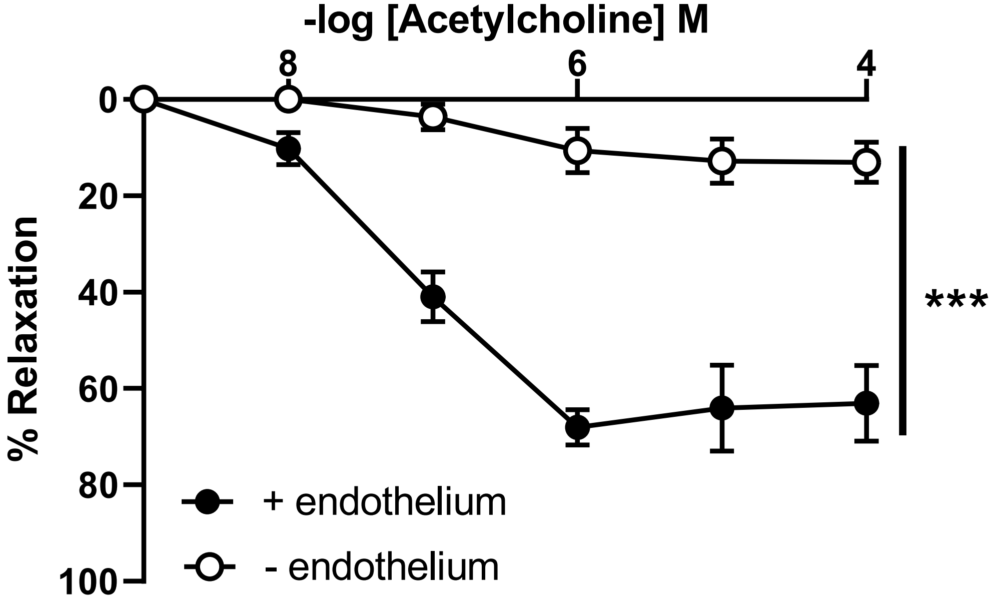

Supplementary Figure 2. Influence of the endothelium on acetylcholine relaxations. Concentration-response curves to acetylcholine in the absence (-) and presence (+) of endothelium in U46619-pre-contracted aortic rings from young OF1 mice. Results are mean ± SEM from n = 5 mice. *** $P < 0.001$ by two-way repeated measures ANOVA with Bonferroni's post hoc test.

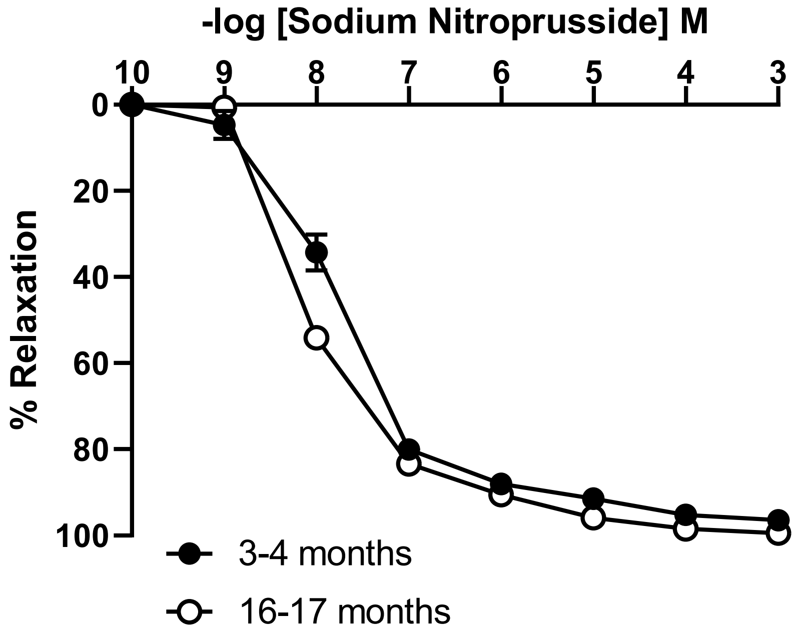

Supplementary Figure 3. Concentration-response curves to sodium nitroprusside in U46619-precontracted aortic rings from 3- to 4-month-old and 16- to 17-months-old C57BL/6 mice. Results are mean ± SEM from n = 3-5 mice.

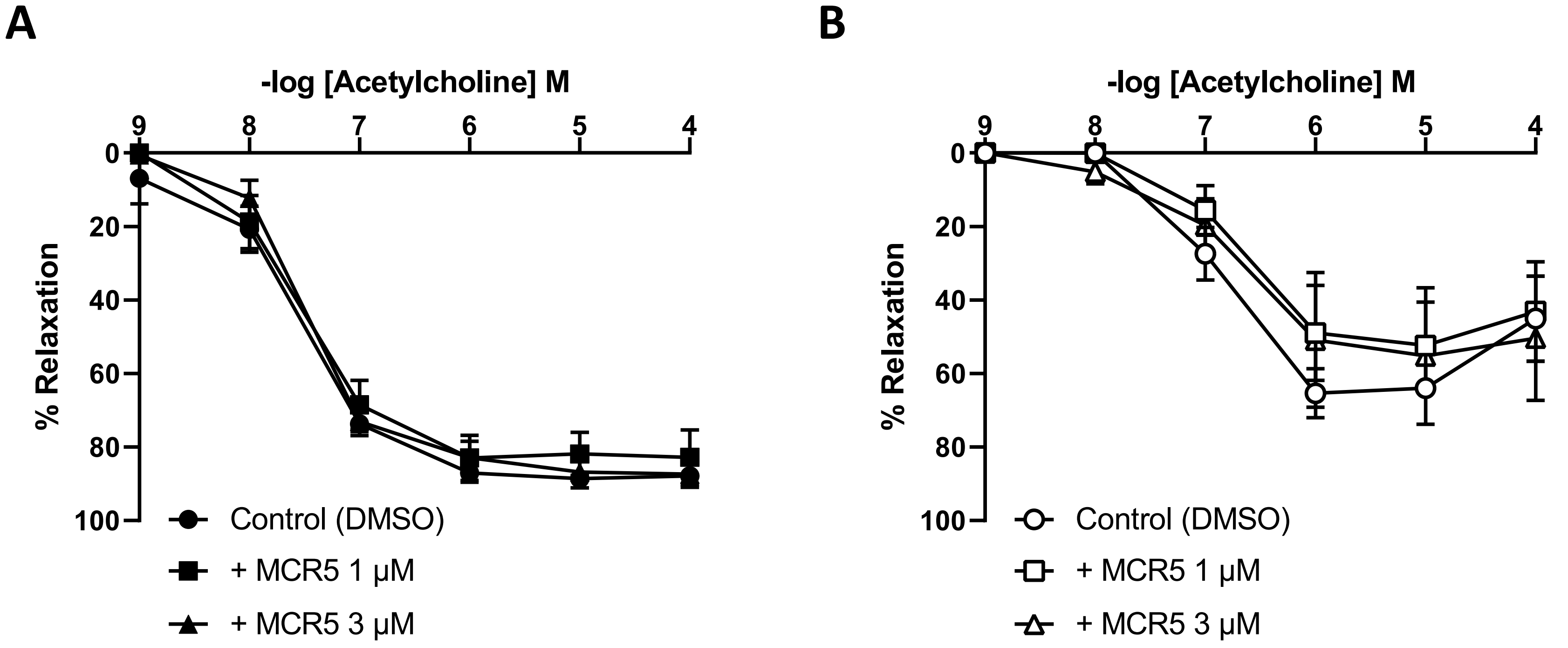

Supplementary Figure 4. Influence of MCR5 (1-3 μM) incubation on aortic acetylcholine relaxations of young and old C57BL/6 mice. Concentration-response curves to acetylcholine in U46619-precontracted aortic rings from 3- to 4-month-old (A) and 16- to 17-months-old (B) C57BL/6 mice. Results are mean ± SEM from n = 5 (A) and 3-4 (B) mice.
